# Behaviour of dissolved inorganic salts in the cooling water of a nuclear power plant open recirculation system and formation of water discharge

**DOI:** 10.1098/rsos.240492

**Published:** 2024-08-07

**Authors:** Olha Biedunkova, Pavlo Kuznietsov, Volodymyr Gandziura

**Affiliations:** ^1^ National University of Water and Environmental Engineering, Rivne, Ukraine; ^2^ Taras Shevchenko National University of Kyiv Institute of Biology and Medicine, Kyiv, Ukraine

**Keywords:** water treatment, maximum permissible concentration and discharge, regression relationships, return and cooling water, cycle of concentration

## Abstract

The main problem in the operation of nuclear power plants (NPPs) is the scale formation of mineral impurities in an open recirculating system (ORS). The discharge of water from an ORS into natural water bodies can alter the chemical equilibrium of wastewater components, necessitating continuous monitoring. The purpose of this study was to analyse the behaviour of dissolved inorganic salts (DIS) in water within an ORS during water treatment, using the Rivne Nuclear Power Plant (RNPP) as a case study. Moreover, the analysis impact of their discharge with return water in the Styr River. The DIS concentration has a significant impact on the efficiency of the system and the environmental of an ORS power plant. Altogether, each of the DIS components was analysed separately using the standard measurement methods, statistical methods of data processing and correlation analysis. In addition, the annual discharge of the DIS components was calculated, and the amount of discharge was assessed for compliance with the maximum discharge limit. Thus, the impact of the formation of DIS and the variations in their concentration levels upon the discharge of wastewater into a natural water body were examined.

## 1. Introduction

The main industries that use an open recirculating system (ORS) for cooling are energy, metallurgy, chemical and petrochemicals, and food processing. Operational water use accounts for the majority of natural water consumed by contemporary industries, including the energy sector [1]. Reliable electricity sources are necessary in the modern world to maintain critical infrastructure [2]. Heat power plants generate most of the electricity in many countries. Despite many efforts intended to improve their operation (because their efficiency has both economic and ecological effects), there are still many environmental issues. Under these circumstances, nuclear power has become the priority electricity source for many countries. However, nuclear power plants (NPPs) also emit environmental pollution, similar to that of any other industry [3]. There are gaseous, liquid and solid emissions from NPPs. Obviously, such emissions include radioactive and non-radioactive substances. In Ukraine, more nuclear units at existing NPPs are projected to have a total capacity of up to 22 GW [4]. Similar strategies are accepted in the USA, China, India and many other countries [1–4]. National safety, including reliable energy supply, is always of the highest priority.

Water is an essential component for the operation of NPPs, as it is needed for cooling in the processes of the steam–water cycle [5]. In an ORS, the same water is used repeatedly to cool the process equipment [6]. The main serious problem in the operation of an ORS is the scale formation of mineral components. Moreover, the formation of scale increases the hydraulic resistance within the system, leading to higher power consumption during its operation. The quantity of make-up water and blow-down water from the ORS required to maintain water quality is constrained by both economic and environmental considerations [7]. However, wastewater discharge from ORS into surface water bodies can lead to changes in the chemical equilibrium of wastewater components, which is a potential technogenic hazard and requires constant monitoring in the operation of NPPs [8]. The performance of chemical processes that determine the scale of formation of mineral components can be strongly affected by different ORS operating conditions, so there is a need for the integration of chemical processes and ORS to use cooling water efficiently and improve the performance of technological processes. A few anions, bicarbonate (HCO_3_^−^), carbonate (CO_3_^2−^), sulfate (SO_4_^2−^) and chloride (Cl^−^), and a few cations, calcium (Ca^2+^), magnesium (Mg^2+^) and sodium (Na^+^), constitute the largest fraction of dissolved inorganic salts (DIS) in the surface water bodies and are important parameters for monitoring chemical processes for ORS. The significance of DIS components on the system performance in ORS is shown in table 1. Therefore, in water treatment technologies for an ORS in NPPs, the components of DIS must meet a number of environmental standards [9], which is also essential for the sustainable development of the entire energy sector [10].

**Table 1.** Sampling frequency of the components of DIS and its significance for system performance. TDS = total dissolved solids, TH = total hardness, TA = total alkalinity.

indicators	significance	removal	sampling frequency
TDS	increasing the content has an impact on the scale formation and water discharge	lime softening, ion exchange, demineralization, reverse osmosis, electrodialysis and desalination	daily
TH and Σ(Ca^2+^, Mg^2+^)	main source of the scale formation CaCO_3_, CaSO_4_ and Mg(OH)_2_		daily
TA and Σ(HCO_3_^−^, CO_3_^2−^)	main source of CaCO_3_, Mg(OH)_2_ scaling and influences corrosion		daily
рН	determines alkalinity or acidity and affects corrosion	acid or alkali addition	daily
SO_4_^2−^	interacts with calcium to form CaSO_4_ scale, causing concrete destruction and corrosion and increases corrosion processes	demineralization, reverse osmosis, electrodialysis and desalination	weekly
Na^+^, Cl^−^	increases corrosion processes		weekly

Heat removal from ORS requires the evaporation of make-up water, and the concentration of DIS in the cooling water increases over time [11], which is defined as the cycle of concentration (COC) [12]. According to [13,14], evaporation processes in ORS occur more intensively with increasing temperature, and the relative value of *W* (1.2) is almost constant for the designed type of cooling tower. With an increase in BD and MU consumption, a greater water exchange is achieved for ORS (1.1) and (1.2), resulting in a decrease in the COC [15].


(1.1) CОС={MU/BD}+W

(1.2) W=Δt⋅K,

where MU is the make-up water rate, m^3^ s^−1^; BD is the blow-down water rate, m^3^ s^−1^; *W* is the windage water in the cooling system; Δ*t* is the temperature difference in the system, °С; and *K* is a coefficient that depends on the air temperature and varies from 0.0012 for 0°C to 0.0016 for 40°C.

**Figure 1.**
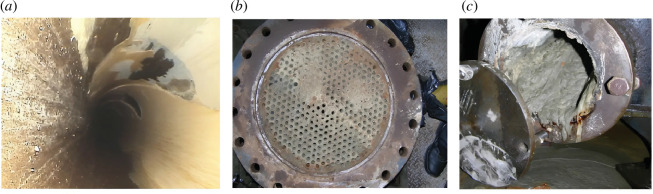
Sediments and sludge of CaCO_3_, CaSO_4_ and Mg(OH)_2_ in ORS: (*a*) scale on condenser tubes, (*b*) mixed deposits in the form of sludge and scale on heat exchanger tubes and (*c*) sludge clogging of the inlet sections of the heat exchanger pipeline.


Once in the ORS, water changes its physicochemical composition under the influence of many factors, and scale formation processes can occur [16], consisting of precipitates of calcium carbonate (CaCO_3_), sulfate (CaSO_4_) and magnesium hydroxide [Mg(OH)_2_] depending on the reaction (1.3)–(1.7) [17,18]. The deposition of CaCO_3_, CaSO_4_ and Mg(OH)_2_ in ORS (figure 1) causes a decrease in the heat transfer of equipment, which reduces the efficiency of NPP operation and increases the operating costs of descaling [2]. Moreover, the presence of scale in the form of sludge can clog the passages of heat exchange equipment and pipelines (figure 1*c*) [19].

(1.3) $$HCO_{3}^{-}↔OH^{-}+CO_{2}$$

(1.4) $${\text{HCO}}_{3}^{-}+{\text{ОН}}^{-}↔{\text{СО}}_{3}^{2-}+{\text{H}}_{2}О$$

(1.5) $$Ca^{2+}\;+\;HCO_{3}^{-}↔CaCO_{3}↓\;+\;H^{+}$$

(1.6) $$Ca^{2+}\;+\;SO_{4}^{2-}↔CaSO_{4}↓$$

(1.7) $${\text{ Mg(НСО}}_{3}{)}_{2}\;+\;{\text{4ОН}}^{-}↔{\text{Mg(OH)}}_{2}↓\;+\;{\text{2СО}}_{3}^{2-}\;+\;{\text{2H}}_{2}О$$

There are several methods for preventing the scale formation: the removal of calcium ions from water during water treatment and the use of anti-scale treatment with a complexing inhibitor and/or the addition of mineral acid [20]. However, a lime softening is the most common method of preliminary water treatment due to the large volumes of water used in NPPs [21]. In a lime softening, calcium bicarbonate Ca(HCO_3_)_2_ and magnesium bicarbonate Mg(HCO_3_)_2_ are converted into calcium carbonate CaCO_3_ precipitates and magnesium hydroxide Mg(OH)_2_, respectively, in the reaction of lime with Ca(OH)_2_ (1.8)–(1.10).


(1.8) $${\text{СО}}_{2}\;+\;{\text{Ca(OH)}}_{2}↔{\text{СаСО}}_{3}↓\;+\;{\text{Н}}_{2}\text{О}$$

(1.9) $${\text{Са(НСО}}_{3}{)}_{2}\;+\;{\text{Са(ОН)}}_{2}↔{\text{2СаСО}}_{3}↓\;+\;{\text{2Н}}_{\text{2}}О$$

(1.10) $${\text{Mg(НСО}}_{3}{)}_{2}\;+\;{\text{2Са(ОН)}}_{2}↔{\text{Mg(OH)}}_{2}↓\;+\;{\text{2СаСО}}_{3}↓\;+\;{\text{Н}}_{2}О$$

The precipitate formed during lime softening can remain in suspension and settle slowly due to its small particle size [19], and flocculants and/or coagulants are used to enhance precipitation [22]. Flocculants, mainly polymeric organic compounds, are used to agglomerate the formed particles, and coagulants, mainly inorganic salts of aluminium or iron sulfate, are used to co-precipitate aluminium or iron hydroxides according to reactions (1.11) and (1.12) [23]. The mechanism of action of flocculants and coagulants in lime softening is based on the neutralization of charged particles dispersed in water, with the formation of a precipitate prone to agglomeration and subsequent sedimentation [24,25]. The significance of DIS components on the system performance in ORS is shown in table 1.


(1.11) $$Al_{2}(SO_{4}{)}_{3}\;+\;6HCO_{3}^{-}↔2Al(OH{)}_{3}↓\;+\;3SO_{4}^{2-}\;+\;6CO_{2}$$

(1.12) $$Fe_{2}(SO_{4}{)}_{3}\;+\;6HCO_{3}^{-}↔2Fe(OH{)}_{3}↓\;+\;3SO_{4}^{2-}\;+\;6CO_{2}$$

**Figure 2.**
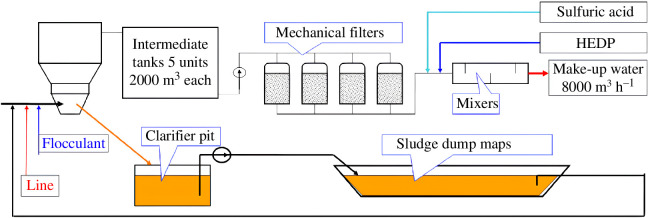
The water treatment of ORS cooling water of the RNPP.

The water treatment of ORS cooling water of a typical NPP on the example of Rivne Nuclear Power Plant (RNPP) is shown in figure 2. Water treatment at RNPP is based on lime softening and flocculant (polyacrylamide), using clarifiers of the VTI−1000 type, and the sludge formed by reactions (1.8)–(1.10) during lime softening is discharged into a sludge pond. The water after liming is treated with sulfuric acid and 1-hydroxyethane-1,1-diphosphonic acid (HEDP) to minimize scale formation.

The relevant documents regulating the environmental impact of water discharge are the Water Code of Ukraine [26] and the Water Framework Directive [27]. The maximum permissible concentrations (MPCs) are presented in [28,29]. Discharge of chemical substances is restricted by taking into account their composition and properties by setting the maximum permissible discharge, which is the maximum permissible rate for a water body from an environmental point of view [30,31], and also by setting limit values (LV) for specific elements in the discharged water. In Ukraine, the discharge of pollutants is regulated by the maximum discharge level (MDL) to [32]. The discharge of cooling water is a special type of water use and is carried out based on permits. Moreover, for each water discharge, MDL values and a list of substances to be monitored in the discharged water should be determined [30–32]. DIS from NPPs pose significant environmental concerns due to their potential impacts on water quality and aquatic ecosystems. NPPs use vast amounts of water for cooling purposes. This water often comes from natural sources like rivers, lakes or the ocean. After being used for cooling, the water is discharged back into these sources. During its use, the cooling water can pick up various DIS from the plant’s cooling system. The RNPP has a permit for special water use [33], and the components of the DIS whose discharge is regulated are Cl^−^ and SO_4_^2−^. High concentrations of DIS, such as chlorides, sulfates and nitrates, can alter the chemical composition of receiving water bodies. This can make the water unsuitable for drinking and other purposes. Understanding the impacts of DIS is essential for NPPs to comply with environmental regulations and discharge permits, which are designed to protect water quality. The environmental assessment of water quality in water bodies of different categories, according to [27,34], is shown in table 2. Conducting comprehensive ecological studies to understand the long-term impacts of DIS on various species and ecosystems can guide better environmental management practices. In summary, the study of DIS from NPPs is critical for protecting water quality and aquatic life. It supports regulatory compliance, fosters environmental sustainability and ensures the responsible development of nuclear energy.

**Table 2.** Classification of water quality of a natural reservoir according to the criteria of salt composition. TDS = total dissolved solids.

indicator	class (І–V), category (1–7) of water quality, mg dm^−3^						
	І ‘high’	II ‘good’		III ‘moderate’		IV ‘poor’	V ‘very poor’
	1	2	3	4	5	6	7
TDS	≤500	501–750	751–1000	1001–1250	1251–1500	1501–2000	>2000
Cl^−^	≤20	21–30	31–75	76–150	151–200	201–300	>300
SO_4_^2−^	≤50	51–75	76–100	101–150	151–200	201–300	>300

The management of cooling water in NPPs is critical for both operational efficiency and environmental protection. Previous research has predominantly focused on the thermal performance of ORS and the mechanical aspects of water recirculation. However, there is a substantial knowledge gap regarding the chemical dynamics of DIS within these systems. Understanding these dynamics is essential as they influence the formation of scale and the overall chemical stability of ORS. In addition, the composition of water discharge from these systems has direct environmental repercussions, particularly in terms of regulatory compliance and ecological impact. Currently, there are no systematic and comprehensive studies on the behaviour of DIS for power plant ORS in the existing literature and this study aims to fill these gaps. This study analyses the behaviour of DIS in an ORS during power plant water treatment using the example of the RNPP, the formation of DIS in ORS effluents and the impact of the discharges on the Styr River water body (Ukraine). The purpose of this study was to analyse the formation of DIS of the RNPP and its influence on changes in DIS in the surface waters of the Styr River to optimize and minimize the ecological impact of discharged water on surface waters and improve the effectiveness of the chemical mode ORS. It is important to study the behaviour of DIS in ORS, as DIS components can have a significant impact on the efficiency of ORS through scale formation and environmental impacts due to the discharge of water containing DIS into a water body. Thus, proper monitoring and understanding of the processes occurring in the ORS cycle and discharge to the water body are critical to ensure safe and efficient operation of ORS in terms of scale formation and conversion of soluble to insoluble salts and compliance with environmental regulations.

Our investigation reveals novel insights into the precipitation and dissolution processes of key salts under varying operational conditions. This includes the identification of critical factors that exacerbate or mitigate scaling and fouling within the system. Furthermore, the study highlights the environmental implications of these processes by analysing the composition and quality of the discharged water, offering new perspectives on compliance with environmental regulations. In summary, this study provides a pioneering exploration of the chemical behaviour of DIS in ORS NPP. The findings offer valuable contributions to optimizing cooling system maintenance, improving operational efficiency and minimizing environmental impacts. This research not only fills a crucial knowledge void but also sets the stage for future studies aimed at enhancing the sustainability of NPP operations.

## 2. Material and methods

This study measured the concentrations of DIS, anions {the sum of HCO_3_^−^ and CO_3_^2−^ [Σ(HCO_3_^−^, CO_3_^2−^)], SO_4_^2−^ and Cl^−^}, cations {the sum of Ca^2+^ and Mg^2+^ [Σ(Ca^2+^, Mg^2+^)] and Na^+^}, as well as the total parameters including total dissolved solids (TDS), total alkalinity (TA), total hardness (TH) and pH value. Measurements were conducted on water samples sourced from the Styr River, encompassing both the water intake and discharge zones of the RNPP (figure 2). Water pH was measured using the potentiometric method with multi-meter І−160. Ca^2+^, Mg^2+^ and TH were measured by ethylenediaminetetraacetic acid complexometric titration. Flame photometry has been used for measuring the concentration of Na^+^ and K^+^, and silver nitrate titrate has been used for measuring the concentration of Cl^−^. The method of barium sulfate turbidity has been used for measuring the concentration of SO_4_^2−^. TDS is determined by the gravimetry method. The gravimetry protocol requires that a volume of filtered sample be evaporated to dryness at about 100°C and then dried to a constant weight at 125 ± 5°C. TA and Σ(HCO_3_^−^, CO_3_^2−^) were measured by the titration method. Standard protocols were used for the measurements, as indicated in table 3. The frequency of measurements is presented in table 1, based on the 2022 monitoring results. Water temperature was measured in accordance with MWV 081/12−0311−06 ‘Surface, groundwater and return water. Methodology for temperature measurements’. Water flow measurements were carried out using standard measurement methods (ultrasonic meters and diaphragm-type instruments). The flow velocity was measured using an acoustic flow meter, and the flow was calculated as the product of the cross-sectional area (m^2^) and the average flow velocity (m s^−1^).

**Table 3.** Characterization of methods for measuring the concentration of MI used in the study.

indicator	CI, mg dm^−3^ (unit)	measurement error relative *δ*, % (absolute Δ, unit)	standard protocol in Ukraine	measuring equipment
Cl^−^	7–1500	*δ* = ±20	MWV 081/12-0653-09 Surface, groundwater and return water. Method for measuring the mass concentration of chlorides by the titrimetric method	burette
SO_4_^2−^	50–500	*δ* = ±9	MWB 081/12-0177-05 Surface and treated wastewater. Method for measuring the mass concentration of sulfates by the titrimetric method	
TA and Σ(HCO_3_^−^, CO_3_^2−^)	5–200	*δ* = ±10	SEA 085-66-82 Unified methods for water quality research	
Na^+^	1–200	*δ* = ±10	ISO 9964-3:1993 Determination of sodium and potassium content by the flame emission atomic spectrometry	flame-photometric liquid analyser
TH and Σ(Ca^2+^, Mg^2+^)	10–150	from 10 to 50: *δ* = ±10; from 50 to 150: *δ* = ±5	MWB 081/12-0006-01 Surface and treated wastewater. Method for measuring the mass concentration of calcium and magnesium by the titrimetric method	burette, laboratory balance
TDS	50–10 000	*δ* = ±5	MWB 081/12-0109-03 Surface, groundwater and return water. Method for measuring the mass concentration of dry residue (dissolved solids) by the gravimetric method	laboratory balance, tumble dryer
pH	(1–10)	(Δ = 0.1)	MWV 081/12-0317-06 Method for measuring the hydrogen pH by the electrometric method	pH meter, рН-measuring and standard electrodes, buffer solutions

Water treatment of the ORS cooling water of the RNPP was carried out by lime softening, followed by stabilization treatment with sulfuric acid and HEDP (figure 2). Lime softening is carried out using lime (CaO) in clarifiers with TH value of 2.2 mmol dm^−3^. Owing to the fact that this process is carried out without heating, TH value of less than 2.2 mmol dm^−3^ is not achieved in the winter, which is a limitation of the descaling method. Acidification with sulfuric acid is carried out to pH 7.4–7.6 [23], and HEDP is dosed variably according to [2]. The raw water requirements for the cooling water circuit for the ORS RNPP are approximately 6000 m^3^ h^−1^ for different operating modes, and the water is discharged into the Styr River at a constant flow rate of approximately 1000 m^3^ h^−1^. The ORS RNPP is designed for operation at COC ranging from 1.5 to 7.0. The DIS discharge (*D*, *t*) for each component was calculated using the formula (2.1), and the annual discharge was calculated according to the annual amount of discharged cooling water.


(2.1)  D=C⋅F/1000000,

where *C* is the concentration of components of DIS in the return cooling water (mg dm^−3^), and *F* is the amount of return cooling water (m^3^).

**Figure 3.**
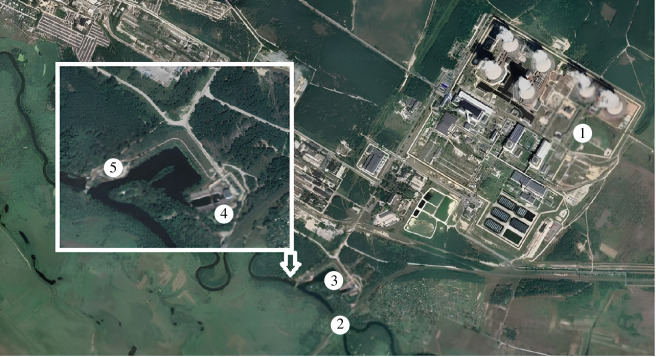
A section of the Styr River in the RNPP water intake and discharge zone (1, plant site of the RNPP; 2, Styr River; 3 and 4, water intake; 5, water discharge).

To assess the discharge quality indicators, the regulated content values of DIS in the cooling water of ORS at NPPs were used in accordance with table 4. In addition, the regulated typical LV and the regulated LV according to table 5 were used. The statistical processing of the research results involved determining the range of data series (min–max), arithmetic mean (*M*), standard deviation (±s.d.), Pearson coefficient (*r*), significance of the connection (*p*) of the appropriate sample and the factor analysis of the data was carried out using standard methods of mathematical statistics with the software package Minitab software (v. 21.4.1, Minitab, LLC) (figure 3).

**Table 4.** Normalized DIS values with respect to the total DIS parameters in the system cooling water.

indicators	regulated content values			
	[35]	[6]	[36]	[37]
pH, unit	6.0–8.5	6.0–8.0	6.0–8.5 at 25°C	6.0–8.5 at 25°C
ТН, mmol dm^−3^	no more than 5.0	no more than 1.20	no more than 7.0	no more than 7.0
TA, mmol dm^−3^	0.8–1.20 without inhibitor and 1.20–1.60 with inhibitor	no more than 1.20	no more than 1.20	no more than 2.5 without inhibitor and no more than 6.0 with inhibitor
Cl^−^, mg dm^−3^	no more than 400	no more than 200	no more than 100	no more than 150
SO_4_^2−^, mg dm^−3^	depending on the quality of concrete structures less than 250 or less than 1500	no more than 250	no more than 500	no more than 500
TDS, mg dm^−3^	no more than 1000	no more than 1350	no more than 1000	no more than 800
Na^+^, mg dm^−3^	no more than 500	not normalized	not normalized	not normalized

**Table 5.** Regulated typical LV and regulated LV for the RNPP in return water.

indicators	typical LV [6]	LV for RNPP [33]
pH, unit	6.0–10.0	6.5–9.0
TSD, mg dm^−3^	–	1000
Cl^−^, mg dm^−3^	–	150
SO_4_^2−^, mg dm^−3^	1000	425

## 3. Results and discussion

**Figure 4.**
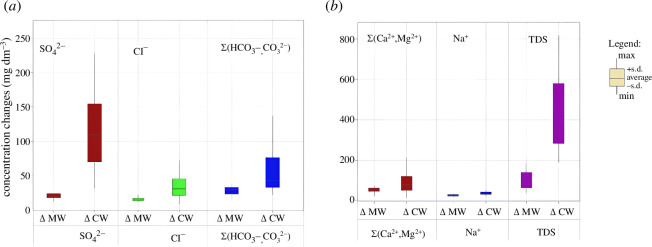
Changes in the concentration of DIS components in the ORS RNPP process cycle, where ΔMW is the difference in the content of DIS components in the Styr River water and make-up water; ΔCW is the difference in the content of DIS components in make-up water and system cooling water (*a*) for DIS anions, (*b*) for DIS cations and TDS.

The make-up water and system water quality parameter were analysed using a *t*‐test for a range of values within the 95% confidence interval (table 4). Thus, the pH, TDS and Cl^−^ and Na^+^ contents in make-up water did not change over time compared with other parameters such as TH and TA. The lime softening water treatment removes Σ(HCO_3_^−^, CO_3_^2−^), Ca^2+^ and Mg^2+^. However, the use of H_2_SO_4_ correction treatment increases SO_4_^2−^ concentration in cooling water (table 4). The make-up water pH was found to be in the range of 7.50–9.51, and the cooling water pH did not significantly vary within this operating range. The effect of make-up water pH on TA was observed over the entire range of the cooling water pH values. The make-up water TDS was in the range of 108–305 mg dm^−3^ and increased proportionally in the system cooling water. The system cooling water pH of the RNPP was within the normal range for most of the time (table 2), with short periods of exceeding the normal range up to pH of 8.55. As a result, the TH of the system’s cooling water did not exceed the standard value on average, with a maximum value of 7 mmol dm^−3^ exceeding the standard value due to the high СОС (1.1). The other system cooling water parameters TA, SO_4_^2−^, Cl^−^, Na^+^ and TDS corresponded to the normalized values in table 2. The changes in the concentration of DIS components in the ORS RNPP process cycle are shown in figure 4*a*. For all components of DIS and TDS, an increase in concentration was recorded, which is due to the processes of evaporation and concentration in the ORS. The concentration of DIS components in the ORS occurs proportionally to the COC [2,26]; however, the formation of sediments and sludge of CaCO_3_, CaSO_4_ and Mg(OH)_2_ can disrupt the proportional relationship between the COC and the concentration of DIS components. This phenomenon, along with the identification of prevailing factors, is subject to the investigation.

**Figure 5.**
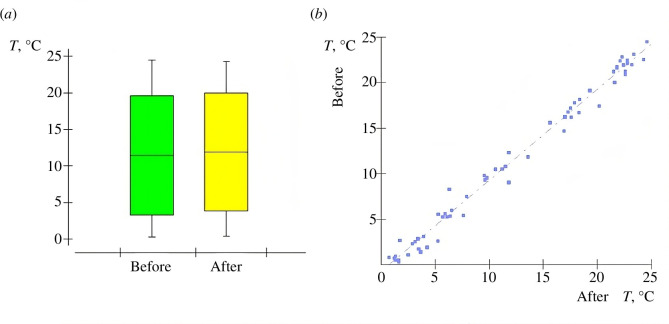
Water temperature of the Styr River before/after the ORS RNPP (*a*) and water discharge and correlation of their values (*b*).

The actual water temperature values of the Styr River before and after the water discharge from the RNPP varied in the range from 0.3 to 24.6°C (figure 5*a*) with *M* = 12.6°C and s.d. = ±8.7°C. The increase in the water temperature of the Styr River (temperature difference) before the intake and after the discharge from the RNPP is observed up to *M* = 1.11°C in the summer season and up to *M* = 2.07°C in the winter season before the RNPP. The established limit for the increase in water temperature, according to the conditions of permit [33], should not exceed 3°C. The temperature difference in the Styr River water before intake and after discharge does not exceed the established limit and has *M* = 1.07°C, with s.d. = ±0.64°C. The correlation between the water temperature values of the Styr River before intake and after the water discharge (figure 5*b*) is positively significant (*p* < 0.0001) at a very strong level (*r* = 0.9934). Higher temperatures in water bodies due to the power plant’s water discharge reduce the oxygen levels in the water, which can affect biodiversity and the trophic network of ecosystems [38]. The change in water temperature of the Styr River in the area of the RNPP intake reflects the known seasonal increase in temperature during the summer period and a decrease in the winter period. The temperature effect of the return water discharge does not exceed the limit. The increase in water temperature in the ORS limits the generating capacity and load of the power plant. Higher cooling water temperatures lead to a reduction in the maximum pressure in the condenser, resulting in decreased turbine efficiency. In addition, at higher ambient water temperatures, the thermal dissipation of heat released at NPP decreases, as warmer cooling water reduces the maximum amount of return water discharged into the natural water body [39].

**Figure 6.**
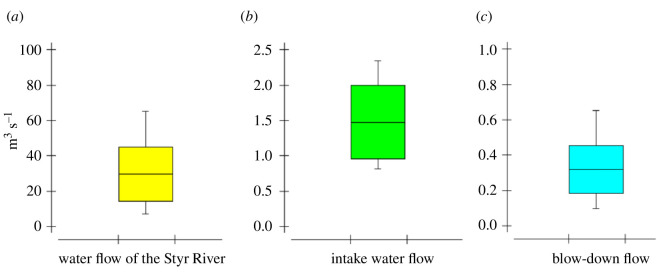
Water discharge from the Styr River (*a*), water intake (*b*) and water discharge (*c*) from the Styr River for the ORS RNPP needs.

Water flows in the Styr River, water intake (MU) and water discharge (BD) from the Styr River for the ORS RNPP needs are shown in figure 6. For the ORS, the MU and BD flows are determined by the cycles of concentration and depend on the load capacity and environmental temperature. For a production capacity of 1000 MW h, the water consumption required for cooling the cooling circuit is 1 m³ s^−1^, of which 0.2–0.4 m³ s^−1^ evaporates and 0.6–0.8 m³ s^−1^ returns to the river [40]. An increase in MU and BD flows is observed during the warm season, which is associated with an increase in ambient temperature and, accordingly, greater water evaporation from the cooling system [9]. The BD and MU flows of the cooling system depend on the temperature; according to [16], evaporation processes occur more intensively with increasing temperature, and with the increase in BD and MU flows, greater water exchange is achieved, resulting in a reduction of the COC.

**Figure 7.**
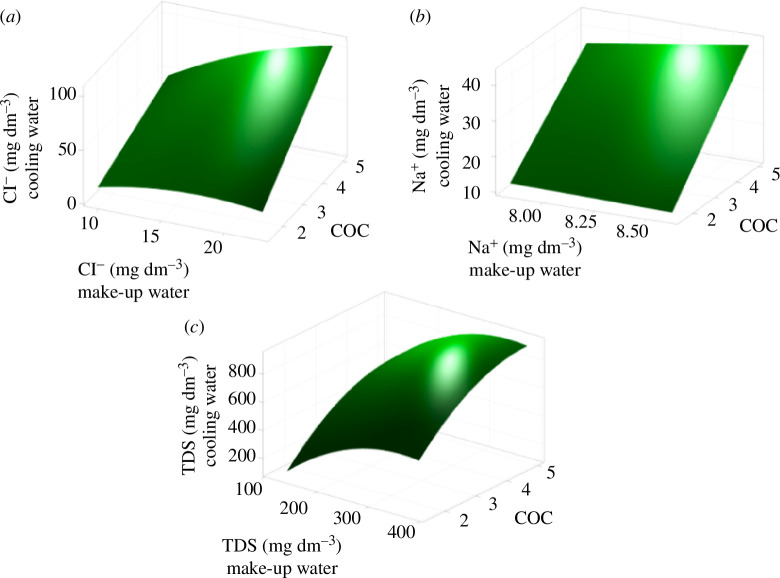
Response surface graphs for (*a*) Cl^−^; (*b*) Na^+^; (*c*) TDS in the make-up and the cooling water depending on СОС of the ORS RNPP.

Compliance with ORS water and chemical regulations requires the implementation of efficient water treatment methods. The process of water treatment with lime softening has a significant potential advantage when used for the treatment of recycled cooling water of ORS, as it achieves lower water consumption and improved water balance [15,19]. However, the cooling water entering the ORS changes its physical and chemical composition under the influence of many factors, which can lead to the formation of carbonate or calcium sulfate (CaSO_4_) deposits with the transformation of DIS into insoluble ones [41]. The make-up water in ORS is concentrated by evaporation and discharged with the return water into a water body, which may affect its ecological status. In addition, corrective treatment for make-up water, in particular the addition of H_2_SO_4_ to neutralize alkalinity, also has a negative impact, increasing the SO_4_^2−^ concentration and requiring increase blow-down of ORS [23]. Response surface methodology is used in a wide spectrum of research activities to design and optimize experimental runs [42]. In practice, figure 7 shows the response surface graphs for Cl^−^, Na^+^ and TDS levels in the system depending on the COC calculated by (1.1). The relationship between the parameters prevails among the make-up water, system cooling water and COC. Moreover, Cl^−^, Na^+^ and TDS are parameters representing the water–chemical regime of ORS, since it does not form a precipitate, and TDS is a general parameter characterizing the ion content in cooling water; among other things, the need for practical research on the formation and changes in the concentration of these indicators in the cooling system is noted [43] (table 6).

**Table 6.** Reported range of values for the make-up and system water quality indicators DIS with their total parameter values for the system cooling water RNPP for 2022.

indications	make-up water				cooling water			
	min	max	aver	±s.d.	min	max	aver	±s.d.
pH, unit	7.50	9.51	9.23	0.61	8.23	8.55	8.35	0.11
ТА, mmol dm^−3^	0,81	1,22	1.05	0.25	2.75	5.25	3.22	1.43
TDS, mg dm^−3^	108	305	244	56	355	750	531	108
ТН, mmol dm^−3^	1.64	2.22	2.05	0,21	2.22	6.53	4.34	1.55
SO_4_^2−^, mg dm^−3^	27.6	101.0	52.12	14.4	80,3	375	144	67.0
Cl^−^, mg dm^−3^	12.2	15.6	13.8	1.9	18.2	80.0	38.6	15.4
Σ(HCO_3_^−^, CO_3_^2−^), mg dm^−3^	48.6	73.2	63.0	9,15	167.8	320.3	196.4	87.23
Σ(Ca^2+^, Mg^2+^), mg dm^−3^	65.6	96.8	82.2	12.4	88.8	261.5	173.6	82
Na^+^, mg dm^−3^	6.25	7.22	6.93	0.31	9.72	28.64	25.14	6.95

The variations observed for the DIS content of the system cooling water of the RNPP ORS are greater than those observed for the make-up water quality, since there is constant make-up and evaporation with concentration [44]. Therefore, the temporal variation is more pronounced in the system cooling water than in the make-up water [45]. Changes in the proportions of make-up water and system cooling water are observed for all the components except TH and Σ(Ca^2+^, Mg^2+^). The contents of TH and Σ(Ca^2+^, Mg^2+^) are underestimated by the proportionality of evaporation calculated from the СOC due to the scale formation with the formation of calcium carbonate and magnesium hydroxide as the system cooling water increases in TA and pH according to the reactions (1.9)–(1.11). Pearson’s correlation coefficient determines the degree of a linear relationship between any two variables on a scale of −1 (perfect inverse relation) to 0 (no relation) to +1 (perfect sympathetic relation). The Pearson correlation matrix (table 7) was used to study the effect of individual make-up water parameters on the cooling water chemistry conditions.

**Table 7.** Pearson correlation matrix for the make-up (MW) and the system cooling (CW) water quality parameters of the ORS RNPP.

indications		MW						
		pH	TН, Σ(Ca^2+^, Mg^2+^)	TА, Σ(HCO_3_^−^, CO_3_^2−^)	TDS	SO_4_^2−^	Cl^−^	Na^+^
MW	pH	1						
	TН, Σ(Ca^2+^, Mg^2+^)	−0.75	1					
	TА, Σ(HCO_3_^−^, CO_3_^2−^)	0.94	0.67	1				
	TDS	0.22	0.58	0.13	1			
	SO_4_^2−^	0.34	0.11	0.05	0.78	1		
	Cl^−^	0.10	−0.09	0.20	0.91	0.54	1	
	Na^+^	0.65	0.55	0.24	0.94	0.68	0.83	1
indications		MW						
		pH	TН, Σ(Ca^2+^, Mg^2+^)	TА, Σ(HCO_3_^−^, CO_3_^2−^)	TDS	SO_4_^2−^	Cl^−^	Na^+^
MW	pH	0.85						
	TН, Σ(Ca^2+^, Mg^2+^)	0.65	0.94					
	TА, Σ(HCO_3_^−^, CO_3_^2−^)	0.99	0.23	0.92				
	TDS	0.13	0,68	0,55	0.98			
	SO_4_^2−^	−0.26	0.05	0.12	0.91	0.93		
	Cl^−^	0.81	0.89	0.80	0.98	0.84	1.00	
	Na^+^	0.80	0.82	0.80	0.95	0.81	1.00	1.00
indications		CW						
		pH	TН, Σ(Ca^2+^, Mg^2+^)	TА, Σ(HCO_3_^−^, CO_3_^2−^)	TDS	SO_4_^2−^	Cl^−^	Na^+^
CW	pH	1						
	TН, Σ(Ca^2+^, Mg^2+^)	0.68	1					
	TА, Σ(HCO_3_^−^, CO_3_^2−^)	0.92	0.57	1				
	TDS	−0.11	0.48	0.18	1			
	SO_4_^2−^	0.05	−0.22	0.37	0.81	1		
	Cl^−^	0.03	0.11	0.15	0.95	0.34	1	
	Na^+^	0.58	0.68	0.55	0.91	0.78	0.86	1

However, it was observed (table 7) that the make-up water рН has an inverse relationship with TH and Σ(Ca^2+^, Mg^2+^), as increasing pH causes an increase in lime dosage using lime softening water treatment and lower TH and calcium levels, and system cooling water pH already has a direct relationship with increasing pH, TH and Σ(Ca^2+^, Mg^2+^). However, the make-up water parameters exhibit strong positive correlation (*r* = 0.9–1.0) between TH and Σ(Ca^2+^, Mg^2+^), as well as between pH and ТА, and between TDS, Cl^−^ and Na^+^. Similar relationships are observed for the system cooling water. The correlations at the average level (*r* = 0.7–0.9) show a negative trend in the make-up water, whereas a positive correlation in the system cooling water is observed between pH and ТА, and Σ(Ca^2+^, Mg^2+^). Thus, the make-up water parameters fixed are positively correlated with the strong levels (*r* = 0.9–1.0) between TH and Σ(Ca^2+^, Mg^2+^); рН and ТА; TDS, Cl^−^ and Na^+^ similar relationships are observed for the system cooling water. The correlations at the average level (*r* = 0.7–0.9) are a negative in the make-up water, and the positive correlation in the system cooling water is between pH and ТА, Σ(Ca^2+^, Mg^2+^). The correlations between make-up water and the system cooling water parameters show a strong positive correlation between TA and pH; TH and Σ(Ca^2+^, Mg^2+^); Cl^−^, Na^+^ and TDS. The system cooling water Cl^−^, Na^+^ concentrations and TSD are influenced by the make-up Cl^−^, Na^+^ concentrations and TDS. The strong correlation between the make-up water and the system cooling water (*r* = 0.9–1.0) between TH and Σ(Ca^2+^, Mg^2+^); pH and TA; TDS, Cl^−^ and Na^+^ is explained by the determining influence of these parameters on the overall indicator (table 7).

**Figure 8.**
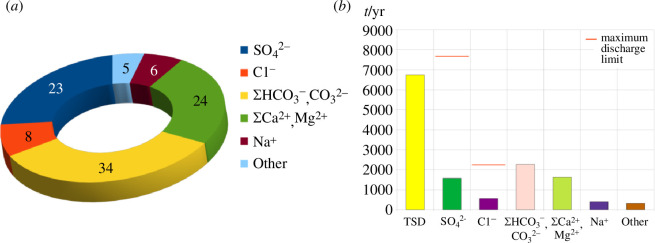
The actual discharge of DIS return cooling water from the RNPP in 2022. (*a*) Percentage of discharge components in relation to TSD, (*b*) discharge amount of DIS.

Among the components of the DIS discharge of the RNPP return cooling water, up to 58% are dominated by Σ(HCO_3_^−^, CO_3_^2−^) and Σ(Ca^2+^, Mg^2+^) (figure 8), and an increase in up to 23% of SO_4_^2−^ is observed due to the corrective treatment of make-up water with H_2_SO_4_. The values of the actual concentrations of DIS in the cooling water do not exceed the values of the typical LV and LV for the RNPP for DIS components in the water discharges of the RNPP, which are regulated (table 3). The actual annual discharge is significantly lower than the MDL and accounts for 20 and 25% of the MDL values for Cl^−^ and SO_4_^2−^, respectively (figure 3*b*). Thus, the values of the actual DIS concentrations and general indicators characterizing the DIS content in the water of the Styr River after the discharge of ORS effluent from the RNPP did not exceed the MPC [28,29,46,47] in the water body (table 8). The classification of water quality (tables 2 and 6) of a natural reservoir according to the criteria of salt composition identifies the water of the Styr River in the zone of influence of RNPP discharges as class I, category 1 and water quality ‘high’. The disruption of the proportional relationship between COC and the concentration of DIS components due to the formation of sediments and sludge indicates a complex interplay of chemical reactions. The precipitates of CaCO_3_, CaSO_4_ and Mg(OH)_2_ not only reduce the concentration of these ions in the solution but also alter the overall ionic strength and saturation levels of other components. This can lead to secondary precipitation reactions, influencing the long-term stability and distribution of these substances in the aquatic environment [48,49].

**Table 8.** Values of actual DIS concentrations and general indicators characterizing the DIS content in the Styr River water after discharge of ORS from the RNPP effluent (2022).

indicators	min–max	*M*	±s.d.	МРС
pH, unit	7.85–8.50	8.19	0.25	6.5–8.5 [28,29,46,47]
TDS, mg dm^−3^	230–550	384	69	1000 [46,47]
ТА, mmol dm^−3^ [Σ(HCO_3_^−^, CO_3_^2−^), mg dm^−3^]	1.86–3.29	2.79	0.65	4.9–6.5 [28,46,47] (300–400) [46,47]
Cl^−^, mg dm^−3^	9.30–23.6	14.8	2.9	300 [29]
SO_4_^2−^, mg dm^−3^	17.3–77.2	41.7	12.5	100 [29]
TH, mmol dm^−3^	2.49–6.65	6.05	1.32	5.0–7.0 [46,47]
Na^+^, mg dm^−3^	7.85–9.66	8.23	0.5	50^a^ [46,47]

^a^ Note: the sum of sodium and potassium ions (Na^+^ + K^+^).

**Figure 9.**
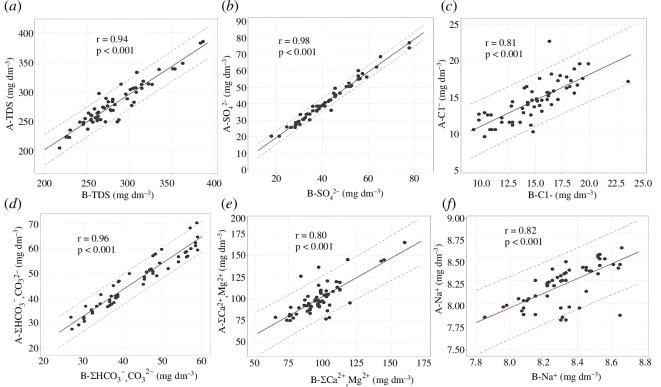
Regression relationships for DIS concentrations in the Styr River water before intake (A−) and after discharge (B−) of the ORS RNPP in 2022. (*a*) TDS; (*b*) SO_4_^2−^; (*c*) Cl−; (*d*) Σ(HCO_3_^−^, CO_3_^2−^); (*e*) Σ(Ca^2+^, Mg^2+^); (*f*) Na^+^.

Multiple linear regression was used as a statistical technique to determine factors that contributed to control indicators [42]. Correlations between DIS for Styr River water samples collected before (A−) and after (B−) the RNPP discharge were estimated (figure 4). However, correlations between DIS components for Styr River (figure 4) show models with high acceptance and a perfect positive correlation between the control parameters (*r* > 0.8) with a significance level of *p* < 0.001. Thus, аnalysing the regression dependencies (figure 9), it can be argued that the discharge has no impact on the water quality indicators of the Styr River, since the results of monitoring DIS components for the Styr River before (A−) and after (B−) the RNPP discharge correlate with each other. A strong positive correlation of DIS was observed for the results of the control before water intake and after water discharge from the RNPP (figure 4), which may indicate the absence of impact of DIS discharges and a significant contribution of the technological aspect to the impact of ORS discharges from the RNPP. Thus, based on the results of the discharge assessment, the chemical regime management of the ORS RNPP for Cl^−^, Na^+^ and TDS did not exceed the typical LV and LV for the RNPP in the return water, water discharge MDL and MPC in the water Styr River. This allows for effective management and rational use of water resources [50,51]. Our findings indicate that the discharge of DIS from the ORS NPP can significantly impact the chemical equilibrium of the receiving water bodies. The increased concentrations of DIS and TDS due to evaporation and concentration processes suggest that these water bodies may experience heightened levels of these components over time. This escalation can lead to several ecological and environmental consequences, including the potential for eutrophication, alteration of water hardness and disruption of aquatic life. Comparing our results with similar studies reveals consistent patterns in the behaviour of DIS components under high evaporation and concentration conditions. For instance, research conducted by Kuznietsov [51,52] found that in power plant cooling systems, the concentration of similar components also increased, leading to substantial changes in the receiving waters’ chemical composition. This study emphasized the importance of monitoring and regulating DIS discharge to mitigate adverse environmental effects. For example, it has set specific limits on the concentrations of various chemicals and sediments permissible in discharge waters [6,33,35–37]. Our findings highlight the necessity for the RNPP to ensure that its discharge levels remain within these limits to prevent environmental degradation.

Understanding the conditions under which these precipitates form and their subsequent impact on water chemistry is essential for developing strategies to manage and mitigate their effects. In addition, exploring the use of advanced water treatment technologies to reduce DIS concentrations before discharge could prove beneficial. In conclusion, the implications of our findings underscore the critical need for stringent monitoring and regulatory measures to control DIS discharge from the ORS RNPP. By comparing our results with existing studies and adhering to established standards, we can better understand and mitigate the environmental impacts, ensuring the protection and preservation of aquatic ecosystems.

## 4. Conclusion

The formation and changes in the composition of DIS as a result of water treatment (lime softening and corrective treatment) were studied, and it was shown that SO_4_^2−^ will be the limiting ion in terms of concentration when using corrective treatment with H_2_SO_4_, and lime softening processes will be determinants of the content of HCO_3_^−^, CO_3_^2−^ and Ca^2+^, Mg^2+^ of the RNPP in the make-up water. The system cooling water TH did not exceed the standard value on average, and it did exceed the standard value of 7 mmol dm^−3^ due to the maintenance of a high COC. Moreover, when cooling water is used in ORS, the concentration of DIS is affected by evaporation, which causes the concentration of DIS components and the total indicators characterizing DIS content to increase proportionally to СOC. The correlation between DIS component values in the make-up and system cooling water of the ORS RNPP was studied. The positive correlation with the strong levels (*r* = 0.9–1.0) between TH and Σ(Ca^2+^, Mg^2+^); рН and ТА; TDS and Cl^−^, Na^+^ are observed for the system cooling water. Therefore, it is explained by determining the influence of DIS components on total indicators characterizing DIS (ТА, ТН and TDS). In addition, the chemical composition of the DIS in the effluent of the ORS RNPP is formed by the input values of the Styr River water (Cl^−^ and Na^+^), water treatment (HCO_3_^−^, CO_3_^2−^, Ca^2+^, Mg^2+^ and SO_4_^2−^) and DIS concentration during evaporation. The study evaluates the impact of anthropogenic activities on the water chemistry and the associated potential ecological risk in the Styr River, specifically in the area influenced by the RNPP water discharge. This assessment is based on the following regulated values: the LV in the return water, the MDL in the water discharge and the MPC in the water body for the components of DIS and general indicators characterizing the DIS content. In water discharges from the RNPP, up to 58% are dominated by HCO_3_^−^, CO_3_^2−^ and Σ(Ca^2+^, Mg^2+^), and DIS concentration in water Styr River did not exceed MPC, water discharge accounts for 20 and 25% of the MDL values for Cl^−^ and SO_4_^2−^, respectively. In practice, the actual values of anthropogenic activities on the DIS discharge did not exceed the established standardized values. In general, the studies indicate that the discharge of DIS into the effluent from the RNPP does not affect the surface waters of the Styr River.

## Data Availability

Data is available online [53].
